# KI Essence extract (a spleen-tonifying formula) promotes neurite outgrowth, alleviates oxidative stress and hypomyelination, and modulates microbiome in maternal immune activation offspring

**DOI:** 10.3389/fphar.2022.964255

**Published:** 2022-08-25

**Authors:** Gilbert Aaron Lee, Hong-Wei Zhao, Yu-Wei Chang, Chia-Jung Lee, Yu-Chen S. H. Yang, Ying-Chieh Wu, Wan-Li Lin, Yun-Ru Liu, De-Shan Ning, Sung-Hui Tseng

**Affiliations:** ^1^ Department of Medical Research, Taipei Medical University Hospital, Taipei, Taiwan; ^2^ Department of Microbiology and Immunology, School of Medicine, College of Medicine, Taipei Medical University, Taipei, Taiwan; ^3^ Child Development Research Center, Taipei Medical University Hospital,, Taipei, Taiwan; ^4^ Infinitus (China) Company Ltd, Guangzhou, Guangdong, China; ^5^ PhD Program for Clinical Drug Discovery of Herbal Medicine, College of Pharmacy, Taipei Medical University, Taipei, Taiwan; ^6^ Graduate Institute of Pharmacognosy Science, College of Pharmacy, Taipei Medical University, Taipei, Taiwan; ^7^ Joint Biobank, Office of Human Research, Taipei Medical University, Taipei, Taiwan; ^8^ Department of Physical Medicine and Rehabilitation, School of Medicine, College of Medicine, Taipei Medical University, Taipei, Taiwan; ^9^ Department of Physical Medicine and Rehabilitation, Taipei Medical University Hospital, Taipei, Taiwan

**Keywords:** mushroom, maternal immune activation, myelination, microbiota, oxidative stress, spleen-tonifying formula

## Abstract

Mushrooms and Chinese traditional herbs have bioactive nutraceuticals with multiple therapeutic functions, including antioxidant and antibacterial activities and microbiome modulation properties. Mushroom-derived bioactive compounds are used in medicines for the treatment of neurological disorders with abnormal brain**–**gut**–**microbiome axis. This study examined the effects of KI Essence extract, a spleen-tonifying formula, on neurite growth, antioxidant activity, hypomyelination modulation, and the microbiome profile in lipopolysaccharide (LPS)-induced maternal immune activation (MIA) offspring. The KI Essence extract induced PC12 cell neurite growth by increasing extracellular signal–regulated kinase (ERK) phosphorylation, promoting 2,2′-diphenyl-1-picrylhydrazyl radical scavenging activity, reducing the level of tert-butylhydroperoxide–induced lipid peroxidation in brain homogenates, protecting PC12 cells from H_2_O_2_-induced cell death (through the inhibition of ERK phosphorylation), alleviating hypomyelination, and downregulating interleukin‐1β through LPS-activated microglia production; moreover, the numbers of Enterobacteriaceae*,* Actinobacteria*,* Peptostreptococcaceae*,* Erysipelotrichaceae, and *Bifidobacterium* bacteria in MIA offspring increased. In summary, the KI Essence extract promotes neurite outgrowth, alleviates oxidative stress and hypomyelination, and modulates microbiota dysbiosis in MIA offspring.

## Introduction

Neurodevelopmental diseases are characterized by an abnormal brain–gut–microbiome axis. The etiopathophysiology of mental disorders involve impaired neurite outgrowth, altered myelination, oxidative stress, and microbial dysbiosis (Graciarena et al., 2018; Nguyen et al., 2018; Xu et al., 2019; Pangrazzi et al., 2020b). Clinical evidence has demonstrated that patients with autism spectrum disorder (ASD) exhibit oxidative stress–related responses, including increases in reactive oxygen species (ROS) and lipid peroxidation levels (Chauhan and Chauhan, 2006; Yui et al., 2020). ROS-induced peroxidation products such as malondialdehyde (MDA), a lipid peroxidation product, can damage cellular components and exacerbate neurodevelopmental disease status (Pangrazzi et al., 2020b; Yui et al., 2020). Oxidative stress also leads to the downregulation of myelin-related gene expression in human oligodendrocytes and of myelin basic protein (MBP) expression levels in the rat brain (Maas et al., 2017). Thus, alleviating oxidative damage is a promising treatment strategy for neurodevelopmental diseases.

Mushrooms and traditional Chinese herbal medicines are considered nutraceuticals that can alleviate the symptoms of neurodevelopmental diseases (Bang et al., 2017). Numerous edible mushrooms, including *Sarcodon scabrosus*, *Ganoderma lucidum*, *Grifola frondosa*, *Hericium erinaceusm*, and *Pleurotus giganteus*, can promote neurite outgrowth in PC12 cells through the extracellular signal–regulated kinase [ERK] signaling pathway (Sabaratnam et al., 2013). The *Tremella fuciformis* extract promotes neurite outgrowth in PC12h cells (Kim et al., 2007). Edible mushrooms (e.g., *Lentinula edodes, Flammulina velutipes*, and *T. fuciformis*) contain bioactive compounds and polysaccharides and thus exhibits antioxidant activity (Li et al., 2014; Yuan et al., 2019; Diallo et al., 2020). The traditional Chinese herbal extract exhibits potent antioxidant activity (Matkowski et al., 2013). In particular, *Lycium barbarum*, *Cassia obtusifolia*, *Euryale ferox*, *Ziziphus jujuba*, and *Prunus mume* extracts exhibit antioxidant activity (Lee et al., 2002; Pi and Lee, 2017; Lu et al., 2019; Wu et al., 2019; Rajaei et al., 2021)*.* The *Crataegus pinnatifida* extract contains maslinic acid, which has been noted to promote synaptogenesis and axon growth through Akt/GSK-3β activation in a cerebral ischemia model (Qian et al., 2015). The medicinal mushroom *Poria cocos* is one of the most commonly used Chinese herbal medicines for autism spectrum disorder (ASD) treatment; studies have verified its anti-inflammatory activity and spleen-tonifying effects (Rios, 2011; Bang et al., 2017; Nie et al., 2020). However, further research is warranted to clarify how the aforementioned mushrooms and traditional Chinese herbal medicines regulate the signaling pathways involved in neuritogenesis and antioxidant responses.

Mitogen-activated protein kinase (MAPK) signaling pathways are involved in the regulation of neuritogenesis and oxidative responses. Specifically, nerve growth factor (NGF) activates ERK 1/2 to promote neuritogenesis (Wang et al., 2011). Oxidative stress also causes the activation of intracellular signaling pathways, including ERK1/2 and p38 MAPK pathways (Rezatabar et al., 2019). The inhibition of these MAPK pathways can protect cells from oxidative stress–induced apoptosis (Rezatabar et al., 2019). Thus, targeting MAPK signaling can inhibit both neuritogenesis and oxidative response–induced cell death.

An increasing number of studies have suggested that gut microbial dysbiosis and oxidative stress play integral roles in neurodevelopmental diseases (Nitschke et al., 2020; Svoboda, 2020). Gut microbiota regulates host physiology, nutrition, and brain function (Vuong and Hsiao, 2017). Dysbiosis is associated with altered integrity of the intestinal barrier and gut inflammation in a maternal immune activation (MIA) model that is known to have features of ASD (Hughes et al., 2018; Li et al., 2021). Microbiota-derived metabolites are correlated with behavioral abnormalities and neuropathology in ASD (Peralta-Marzal et al., 2021), suggesting that gut dysbiosis is associated with ASD pathophysiology.

In traditional Chinese medicine, neurological disorder treatment mainly involves tonifying the spleen and invigorating the brain (Greenwood, 2017). Mushrooms contain bioactive ingredients that modulate gut microbiota and increase spleen Qi (Greenwood, 2017; Ma et al., 2021; Vamanu et al., 2021). KI Essence is a commercial product that contains mushrooms and traditional Chinese herbal extracts; its ingredients have spleen-tonifying effects and can modulate the gut microbiome (Xu et al., 2015; Xu et al., 2021; Zou et al., 2021). MIA elicits oxidative and inflammatory responses during pregnancy, which lead to the development of an abnormal brain–gut–microbiota axis in offspring (Simoes et al., 2018; Lee et al., 2021). In this study, we assessed the potential of the KI Essence extract for neurite outgrowth promotion, oxidative stress alleviation, and maternal infection–induced abnormal brain–gut–microbiota profile modulation in a MIA animal model.

## Materials and mehthods

### KI Essence extract preparation

The KI Essence extract examined in this study was obtained from Infinitus (Guangzhou, Guangdong, China). The raw materials of this extract, including fresh and dry materials, were *Lentinula edodes* (Berk.) Pegler, *Flammulina velutipes* (Curtis) Singer, *Wolfiporia extensa* (Peck) Ginns*, Tremella fuciformis* Berk, *Crataegus pinnatifida* Bunge [Rosaceae], *Lycium barbarum* L. [Solanaceae], *Senna obtusifolia* (L.) [Fabaceae], *Euryale ferox Salisb* [Nymphaeaceae], *Ziziphus jujube* Mill. [Rhamnaceae], *Prunus mume* (Siebold) Siebold & Zucc. [Rosaceae], and *Ostreae gigas*. The weight percentages of each raw material used in the KI Essence extract preparation are presented in Supplementary Table S1. In total, 100 g of raw materials were extracted twice with 1.2 and 1 L of 95°C distilled water for 1.5 and 1 h, respectively. Next, the extract was concentrated in vacuo to obtain a final yield of 24%.

### High-performance liquid chromatography fingerprint analysis

The KI Essence extract (10 mg) was dissolved in H_2_O to obtain a high-performance liquid chromatography (HPLC) sample solution (10 mg/ml). HPLC fingerprint analysis was conducted using a Waters HPLC system (Milford, MA, United States) comprising a Waters 600 pump system, Waters 2996 Photodiode Array Detector, Waters 717 plus Autosampler, and Sugai U-620 Column Oven (Wakayama City, Japan). A Cosmosil 5C18-MS-II reversed-phase column (5 μm, 4.6 mm × 250 mm; Nacalai Tesque, Japan) equipped with a LiChrospher RP-18 end-capped guard column (5 μm, 4.0 mm × 10 mm; Merck, Germany) was used for the stationary phase. The gradient eluents consisted of eluents A (H_2_O:KH_2_PO_4_:10% H_3_PO_4_ = 1,000 g:2.72 g:1 ml), B (acetonitrile), and C (H_2_O), with the following profile: 0–25 min, 80%–100% A and 0%–20% B; 25–45 min, 65%–80% A and 20%–35% B; 45–60 min, 0%–65% A, 35%–80% B, and 0%–20% C; 60–65 min, 10%–80% B and 20%–90% C; and 65–70 min, 0%–100% A, 0%–10% B, and 0%–90% C. The applied flow rate was 1 ml/min, and the column temperature was maintained at 35°C. The relevant data are presented in Supplementary Figure S1. The following components of the KI Essence extract were detected: cytosine, cytidine, hypoxanthine, uridine, guanosine, adenosine, and maslinic acid.

### High-performance liquid chromatography analysis of marker substances in KI essence extract

We used a Hitachi HPLC system, consisting of a Chromaster 5110 pump system, Chromaster 5430 Photodiode Array Detector, Chromaster 5210 plus Autosampler (Hitachi, Japan), and Super CO-150 column oven. A LiChrospher RP-18 reversed-phase column (5 μm, 4.6 mm × 250 mm; Merck) equipped with a LiChrospher RP-18 end-capped guard column (5 μm, 4 mm × 10 mm; Merck) was used as the stationary phase. The mobile phase included 0.05% trifluoroacetic acid (CH_3_CN gradient elution: 0 min, 95:5; 50 min, 0:100). The flow rate was 1 ml/min, and the column temperature was maintained at 40°C. The ultraviolet detection wavelength of maslinic acid was 215 nm. Because of the poor water solubility of maslinic acid, 0.1 g of the KI Essence extract was extracted using 10 ml of 50% methanol through ultrasonic oscillation at 25°C for 20 min to obtain total maslinic acid. The sample was subsequently filtered through a 0.45-μm syringe filter, and a 5-μl aliquot was directly injected into the HPLC system. Maslinic acid standard (Sigma) was used to identify the target peak and amounts of maslinic acid in KI Essence extract. The concentration range of the maslinic acid calibration curve was 10–500 μg/ml. Maslinic acid content in the KI Essence extract was found to be 11.3 ± 0.7 mg/g.

### Phenol–sulfuric acid method for measuring total carbohydrates

We mixed 100 μl of 10 mg/ml KI Essence extract with 800 μL of 95% ethanol with thorough stirring. This mixture was allowed to stand for 30 min and then centrifuged, and the supernatant was discarded. The obtained pellet was washed with 500 μl of 80% ethanol and centrifuged to remove the solvent. The aforementioned steps were repeated three times. Thereafter, the precipitate was dissolved uniformly in 2 ml of 2 M sulfuric acid. Subsequently, 200 μl of phenol and 100 μl of sulfuric acid were added into an Eppendorf vial with the dissolved precipitate. After the solution was reacted for 15 min in a 100°C water bath, glucose (0, 12.5, 25, 50, and 100 μg/ml) was used as a standard for preparing a calibration curve, and optical density (OD) at 480 nm was interpolated to calculate the content of condensed tannins relative to that of glucose (Masuko et al., 2005). The KI Essence commercial product used here contained 70 mg/g of carbohydrates (including monosaccharides, disaccharides, oligosaccharides, and polysaccharides).

### Cell culture

PC12 cells, obtained from American Type Culture Collection (USA; ATCC CRL-1721), were maintained in RPMI 1640 medium (Gibco-Life Technologies, United States) supplemented with nutrient mixture F-12 (Gibco-Life Technologies), 5% fetal bovine serum (FBS), 10% horse serum, and penicillin–streptomycin. The cells were maintained in an incubator at 37°C under atmospheric conditions (CO_2_:air = 5%:95%).

### PC12 cell viability and neurite outgrowth assay

PC12 cells were seeded in six-well culture plates (Corning, NY, United States) coated with 0.1 mg/ml poly-l-lysine at a density of 0.7 × 10^6^ cells per well in culture medium for 1 day. The cultured PC12 cells were then transferred to low-serum medium (containing 1% horse serum with 0.5% FBS) and cultured for 24 h. Subsequently, these cells were stimulated with 50 ng/ml NGF for 2 days in the low-serum medium with various concentrations of the KI Essence extract. The viability of differentiated PC12 cells was assessed through cell counting kit-8 (CCK-8) assays (Dojindo, Japan). OD at 450 nm, which was measured using a spectrophotometer (Thermo Fisher Scientific, USA), was used to estimate cell viability. In the experiments for counting the neurite-bearing PC12 cells, the cells were photographed using a digital camera under a phase-contrast microscope. The images of five randomly selected fields were obtained for each dish, and a mean of 10–20 PC12 cells per field were observed. The percentage of neurite-bearing cells per field was calculated using the following equation: (number of neurite-bearing cells/total number of cells) × 100. Finally, the results from all the fields were tallied and divided by the total number of fields (*n* = 5) to obtain the percentage of neurite-bearing cells per condition (Wiatrak et al., 2020).

### Viability of H_2_O_2_-induced differentiated PC12 cell death assay

PC12 cells were seeded in 96-well culture plates (Corning, United States) coated with 0.1 mg/ml poly-l-lysine at a density of 10,000 cells/well in a culture medium for 1 day. The cultured PC12 cells were then transferred to low-serum medium containing 50 ng/ml NGF and cultured for 2 days. Subsequently, the differentiated PC12 cells were stimulated with 200 µM H_2_O_2_ and various concentrations of the KI Essence extract (0, 0.5, and 1 mg/ml) for 1 day, after which a CCK-8 viability assay was performed (Dojindo).

### Western blotting

We seeded 1 × 10^6^ PC12 cells in 6-cm tissue culture dishes, with overnight incubation. The cultured PC12 cells were then transferred to low-serum medium (containing 1% horse serum with 0.5% FBS) and cultured for 24 h. The PC12 cells were stimulated with 50 ng/ml NGF and the KI Essence extract for 0, 0.5, or 1 h. In another set of experiments, 1 × 10^6^ NGF–induced differentiated PC12 cells were treated with 200 µM H_2_O_2_ and the KI Essence extract for 0, 0.5, or 1 h. Subsequently, cell lysates were prepared using a PRO-PREP protein extraction solution (iNtRON Biotechnology, Korea) containing 2 mM Na_3_VO_4_. The cell lysates were immunoblotted using primary antibodies against p-ERK1/2, p-p38, and β-Actin (Cell Signaling Technology, USA) and horseradish peroxidase–conjugated goat antirabbit or antimouse immunoglobin G (GoalBio, Taiwan). All data were acquired using a ChemiDoc Touch Imaging System (Bio-Rad, USA).

### 2,2′-diphenyl-1-picrylhydrazyl scavenging assay

We added a 100-µL aliquot of 500 µM 2,2′-diphenyl-1-picrylhydrazyl (DPPH)–ethanol solution to each well of a 96-well plate, followed by the addition of 100 μl of the KI Essence extract at various concentrations. Butylated hydroxytoluene was used as a standard antioxidant compound. The plates were incubated for 30 min in the dark, and absorbance was measured at 530 nm on an enzyme-linked immunosorbent assay (ELISA) microplate reader (Thermo Fisher Scientific). Moreover, 300-μl aliquots of ethanol were used as blanks, and the following equation was used to calculate the DPPH radical scavenging rate (%): [1 − (*S*_*T*_/*E*_*C*_)] × 100, where *S*_*T*_ and *E*_*C*_ are the OD at 530 nm of the sample and control, respectively.

### Antiperoxidation effects of KI Essence extract through malondialdehyde assay

Lipid peroxidation levels in brain homogenates were determined by measuring MDA levels. In brief, brain samples from C57BL/6 mice were first homogenized in ice-cold phosphate-buffered saline (PBS) at a concentration of 25% (w/v). The protein levels in the homogenized tissues were quantified using a protein assay dye (Bio-Rad). The brain homogenates (200 μg/ml) were incubated with 50 mM tert-butyl hydroperoxide (t-BHP; Sigma) and various concentrations of the KI Essence extract for 1 h at 37°C. The MDA levels in the sample were measured using an MDA assay kit (Abcam, USA) according to the manufacturer’s instructions.

### Lipopolysaccharide-induced maternal immune activation rat model and oral KI Essence extract treatment

Eight-week-old female Wistar rats (BioLASCO, Taiwan) were mated overnight with male rats; the presence of a vaginal plug was used to confirm the success of mating. Each pregnant rat was allowed to raise its own litter in an individual cage. Subsequently, 500 μg/kg lipopolysaccharide (LPS; from *Escherichia coli* O127:B8; Sigma) or PBS was injected intraperitoneally into the pregnant rats on gestation day 9.5. The 5-week-old male offspring of the rats were treated orally with H_2_O or the KI Essence extract (40 mg/kg) for 2 weeks. Thereafter, their stool and brain were collected for microbiota analysis and immunohistochemistry assay, respectively. The experiments were conducted in accordance with the guidelines of the International Council for Laboratory Animal Science for the care and use of laboratory animals in experiments. Moreover, all animal procedures were approved by the Animal Care and Use Committee of Taipei Medical University (LAC-2019-0198).

### 16S rRNA gene and next-generation sequencing

The detailed procedure for performing 16S rRNA gene sequencing and next-generation sequencing has been provided elsewhere (Lee et al., 2021). The rats were allowed to defecate freely in clean cages, and DNA was extracted from fresh stool samples by using the QIAamp Fast DNA Stool Mini Kit (Qiagen, Germany). Library preparation was performed using an Illumina MiSeq system in accordance with the protocol for 16S rRNA gene amplicons. The universal primers 341F and 805R were used to amplify the V3–V4 region of bacterial 16S rRNA genes. Demultiplexed, paired reads were removed using Cutadapt (version 1.12). The filtered reads were processed using the DADA2 package (version 1.14.1) in R (version 3.6.1) (Callahan et al., 2016a; Callahan et al., 2016b); however, the rarefying procedure was not performed. V3–V4 sequence variants in the samples were inferred using the DADA2 package, and the frequency of each sequence variant in each sample was obtained. Taxonomy assignment was conducted using the SILVA database (version 138) (Quast et al., 2013), with a minimum bootstrap confidence of 80. The multiple sequence alignment of variants and the preparation of a phylogenetic tree were performed using DECIPHER (version 2.14.0) and phangorn (version 2.5.5), respectively (Schliep, 2011). The taxonomy assignment, count table, and phylogenetic tree were applied in a phyloseq object, and community analysis was conducted using phyloseq (version 1.30.0) (Mcmurdie and Holmes, 2013). Alpha diversity indices were calculated to estimate the richness function of the phyloseq package. Statistical analyses were conducted using the Wilcoxon–Mann–Whitney test (*α* = 0.05). UniFrac distances were calculated using the GUniFrac package (version 1.1) to assess community dissimilarity among the groups examined in the present study (Chen et al., 2012). Principal coordinate analysis ordination was applied for UniFrac distances, and the adonis and betadisper functions from the vegan package (version 2.4) for R were used to analyze the dissimilarity of composition among the examined groups and the homogeneity of their dispersion, respectively.

### Immunohistochemistry

The rats were anaesthetized with Zoletil (40 mg/kg) and Xylazine (10 mg/kg), then transcardially perfused with PBS and 4% paraformaldehyde. Their whole brains were fixed with 4% paraformaldehyde for 3 days, and 2-mm coronal slices were embedded in paraffin blocks. These blocks were sliced into 5-µm-thick sections, which were then deparaffinized, rehydrated, and subjected to an antigen retrieval process. Subsequently, the sections were stained with horseradish peroxidase–conjugated MBP antibody (Abcam), followed by staining with 3,3′-diaminobenzidine and hematoxylin, using a Chemicon IHC Select system (Millipore, USA). The sections were observed through microscopy (Olympus/Bx43, Japan), and the MBP-positive area were calculated using the tissue analysis software program HistoQuest (Tissue Gnostics, Austria).

### Interleukin-1β production in lipopolysaccharide-stimulated microglia assay

Enriched glial cultures (microglia and astrocytes) were prepared from the brains of newborn C57BL/6 mice (National Laboratory Animal Center, Taiwan) that were collected on postnatal day 1. In brief, cerebral cortical cells were cultured in Dulbecco’s Modified Eagle Medium/Nutrient Mixture F-12 containing 10% FBS and 1% penicillin–streptomycin for 14 days to enable their differentiation into glial cells. Microglia were detached by shaking the culture flasks containing the cells at 160 rpm for 5 h. The suspended microglia were collected and seeded in 96-well culture plates coated with poly-D-lysine at 5 × 10^4^ cells per well for 1 day. The purity of these isolated cells was measured through staining with CD11b antibody (Biolegend, USA), and these cells were analyzed through flow cytometry. The obtained microglia were stimulated using 250 ng/ml LPS in different concentrations of KI Essence extract for 24 h. The interleukin (IL)-1β content of the culture supernatant was analyzed using a ELISA MAX Deluxe Set Mouse IL-1β kit (Biolegend).

### Statistical analysis

For neurite-bearing cell, MDA content, western blot, MBP^+^ area, IL-1β production data analyses, one-way ANOVA was performed using Prism (GraphPad, USA); in the analysis results, error bars represent the standard errors of the mean. The microbiota enrichment analysis of the groups was conducted using the linear discriminant analysis (LDA) effect size (LEfSe) method. Data were compared using the Kruskal–Wallis and Wilcoxon tests; differences were considered significant when *p* ≤ 0.05 and logarithmic LDA score ≥ 2 (Segata et al., 2011).

## Results

### Effects of KI Essence extract on neurite outgrowth in PC12 cells

Our phenol–sulfuric acid analysis revealed that the KI Essence extract used in this present study contained 70 mg/g carbohydrates (including monosaccharides, disaccharides, oligosaccharides, and polysaccharides; data not shown). The key components of the KI Essence extract include several edible mushrooms, and nucleotides are major components of many edible mushrooms (Ranogajec et al., 2010). As shown in Supplementary Figure S1, several nucleotides were detected in the KI Essence extract, which included cytosine, cytidine, hypoxanthine, uridine, guanosine, and adenosine. Maslinic acid was also detected using HPLC finger printing and HPLC analyses (Supplementary Figures S1,S2).

**FIGURE 1:**
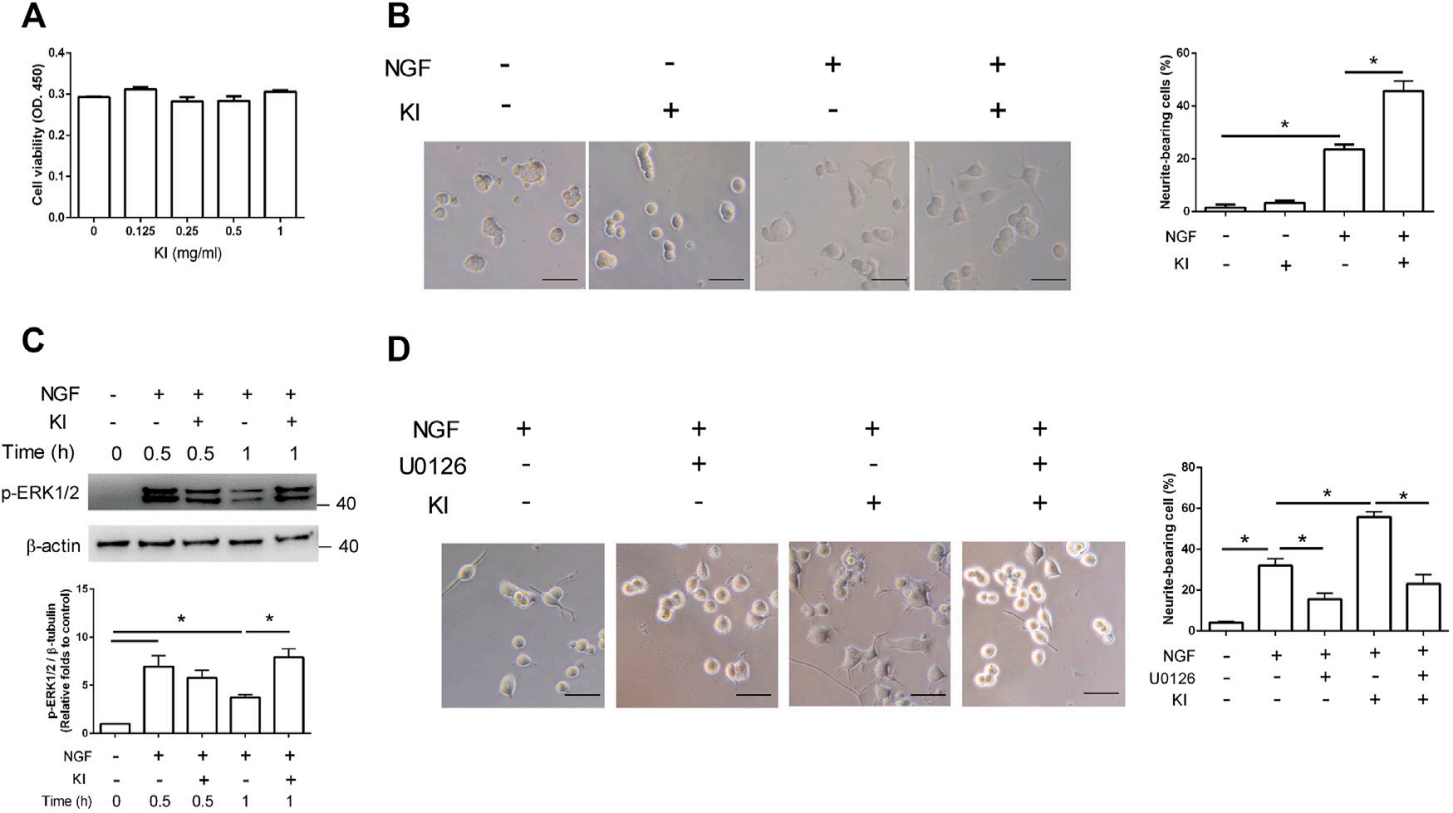
PC12 cell neurite growth induction through extracellular signal–regulated kinase (ERK)1/2 phosphorylation after KI Essence extract treatment. **(A)** KI Essence extract treatment did not affect the viability of PC12 cells. PC12 cells were pretreated with low-serum medium for 1 day and then with various concentrations of the KI Essence extract and nerve growth factor (NGF; 50 ng/ml) for 48 h. PC12 cell viability was determined using cell counting kit-8 (CCK-8) assay. **(B)** Phase-contrast images of cells on day 2 after treatment with NGF in presence or absence of the KI Essence extract (0.25 mg/ml; scale bar: 50 µm). PC12 cells were cotreated with the KI Essence extract and NGF for 2 days, and percentages of neurite-bearing cells on day 2 was assessed. The data are expressed as means ± standard errors of means (SEMs) for (*n* = 3; ^∗^ *p* < 0.05). **(C)** PC12 cells treated with 0.25 mg/ml KI Essence extract for various durations (0, 0.5, and 1 h). Phosphorylation levels of ERK (p-ERK) 1/2 and β-tubulin were analyzed through Western blotting. Quantitation of p-ERK1/2 to β-tubulin is presented in the bar graph (*n* = 3; ^∗^ *p* < 0.05). **(D)** PC12 cells were treated with the KI Essence extract (0.25 mg/ml) and NGF for 2 days in the presence or absence of 10 µM U0126. Phase-contrast images of neurite-bearing cells on day 2 are shown (scale bar: 50 µm). The data are expressed means ± SEMs (*n* = 3). Quantitation of percentage of neurite-bearing cells is presented in the bar graph ^∗^ *p* < 0.05).

We first used the CCK-8 assay to assess whether the KI Essence extract affected PC12 cell viability. The results indicated that treatment with the KI Essence extract at various concentrations did not affect the OD at 450 nm, indicating that this extract did not affect the differentiated PC12 cell viability (Figure 1A). Subsequently, we examined whether the KI Essence extract affected the neuritogenesis of PC12 cells. After 48 h of NGF stimulation, the percentage of neurite-bearing cells increased significantly to 21.5% ± 1.8% in PC12 cells treated with 50 ng/ml NGF compared with the negative control (5% ± 0.5%; Figure 1B). Furthermore, the KI Essence extract increased the percentage of neurite-bearing cells in a dose-dependent manner under NGF stimulation (Figure 1B and Supplementary Figure S3). However, treatment with only the KI Essence extract did not induce neurite outgrowth in PC12 cells. To assess whether KI Essence extract treatment enhanced NGF-induced ERK signaling pathways in PC12 cells, we examined the phosphorylation level of ERK (i.e., p-ERK1/2 level) in PC12 cells. The results indicated that the KI Essence extract enhanced p-ERK1/2 expression in NGF-stimulated PC12 cells (Figure 1C). We next assessed whether the KI Essence extract promoted neurite outgrowth under NGF stimulation through MEK1 and MEK2 activation. Our results indicated that U0126, which inhibits MEK1 and MEK2, abolished the KI Essence extract–induced neurite outgrowth effect in PC12 cells under NGF stimulation (Figure 1D, column 4 vs. 5). Therefore, the KI Essence extract enhanced neurite growth in PC12 cells through ERK1/2 phosphorylation.

### Characterization of radical scavenging and anti-lipid peroxidation properties of KI Essence extract

**FIGURE 2:**
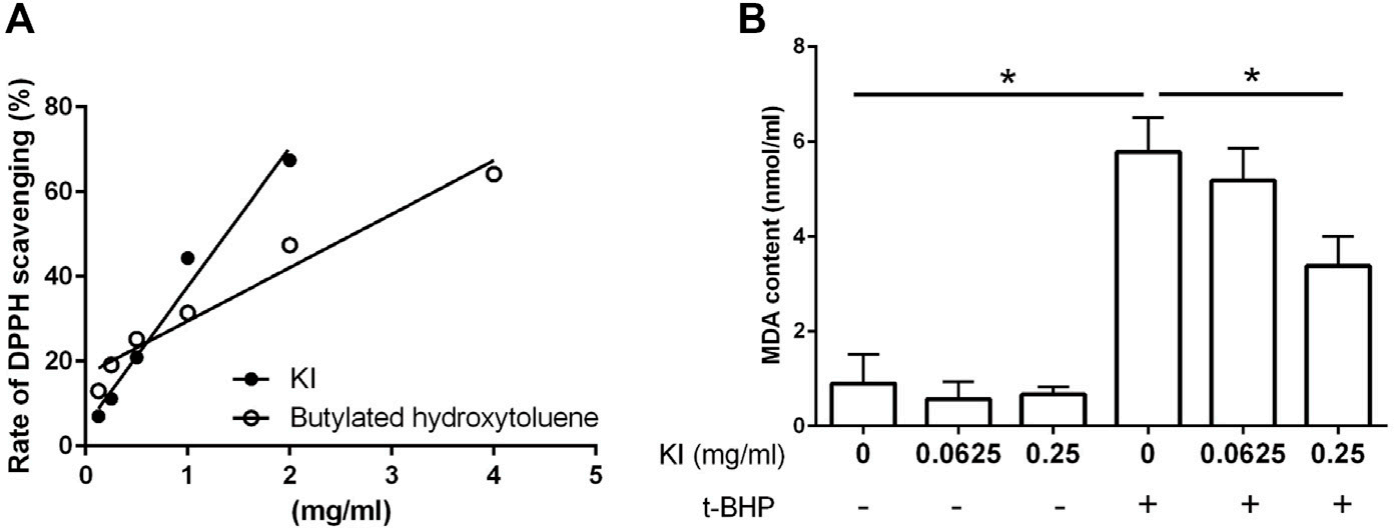
Characterization of KI Essence extract in radical scavenging and anti-lipid peroxidation properties. **(A)** 2,2′-diphenyl-1-picrylhydrazyl (DPPH) scavenging property of the KI Essence extract and butylated hydroxytoluene. 500 µM DPPH were tested at various concentrations of the KI Essence extract and butylated hydroxytoluene. **(B)** Effects of the KI Essence extract on tert-butyl hydroperoxide (t-BHP)-induced lipid peroxidation in brain homogenates. Brain homogenates were incubated with various concentrations of the KI Essence extract (0, 0.0625, and 0.25 mg/ml) with (+) or without (−) t-BHP stimulation for 1 h. Malondialdehyde (MDA) contents are presented in the bar graph. The results are presented as means ± standard errors of means (*n* = 3; *^∗^p* < 0.05).

The radical scavenging property of the KI Essence extract was determined using a DPPH chemical test. As illustrated in Figure 2A, the DPPH radicals were inhibited by KI Essence extract in a dose-dependent manner; it was found to be better than butylated hydroxytoluene, a standard antioxidant compound (Olugbami et al., 2014). Because oxidative stress is a key inducer of central nervous system developmental diseases, we evaluated the effects of the KI Essence extract on t-BHP-induced lipid peroxidation in mouse brain homogenates. The KI Essence extract exerted a considerable, dose-dependent inhibitory effect on MDA production in the homogenates (Figure 2B).

### Effects of KI Essence extract on H_2_O_2_-induced cell death of differentiated PC12 cells

**FIGURE 3:**
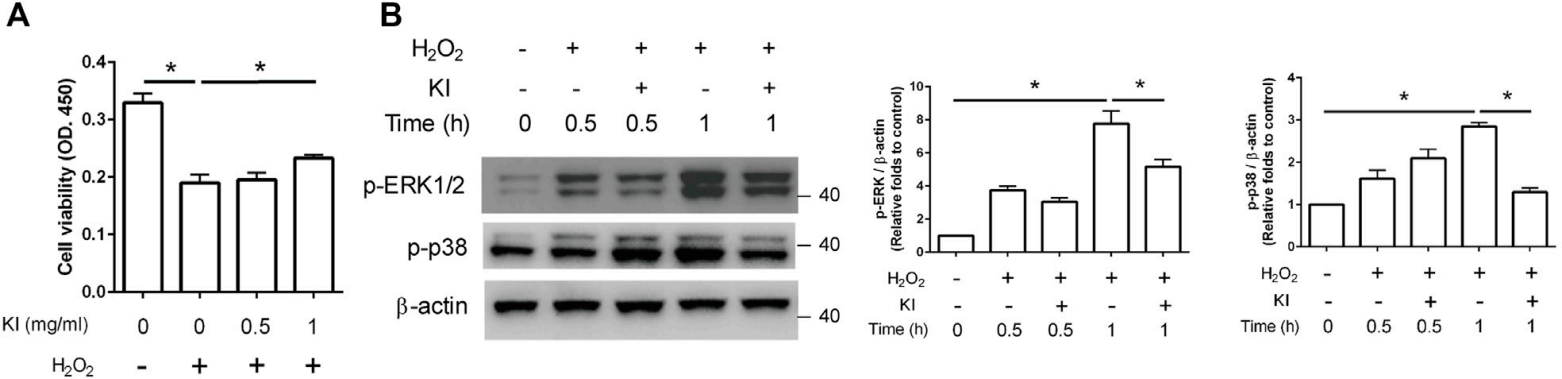
Protective effect of KI Essence extract treatment on PC12 cells against H_2_O_2_-induced cell death through inhibition of ERK phosphorylation. **(A)** PC12 cells were treated with 200 µM H_2_O_2_ and various concentrations of the KI Essence extract for 1 day. Viability of PC12 cells was measured using CCK-8 assay. The results are presented as means ± standard errors of means (SEMs; *n* = 3; *^∗^p* < 0.05). **(B)** PC12 cells treated with 1 mg/ml KI Essence extract for various durations (0, 0.5, and 1 h) in the presence of 200 µM H_2_O_2_. Levels of p-ERK1/2, p-p38, and β-Actin were analyzed through Western blotting. Quantitation of p-ERK1/2 and p-p38 to β-Actin is presented in the bar graph. The results are presented as means ± SEMs (*n* = 3; *^∗^p* < 0.05).

The viability of H_2_O_2_-treated PC12 cells was considerably (62.5%) lower than that of control cells (Figure 3A, column 1 vs. 2). The viability of KI Essence extract–treated cells was higher than that of untreated cells, indicating that KI Essence extract treatment reduced the toxic effect of H_2_O_2_ stimulation (Figure 3A, column 2 vs. 4). We then assessed whether the KI Essence extract affected H_2_O_2_-induced ERK1/2 and p38 signaling pathways in differentiated PC12 cells. The results revealed that H_2_O_2_ treatment significantly upregulated ERK1/2 and p38 phosphorylation after 1 h of stimulation. By contrast, KI Essence extract treatment ameliorated ERK1/2 and p38 phosphorylation (Figure 3B). Therefore, the KI Essence extract protected differentiated PC12 cells from H_2_O_2_-induced oxidative stress and ameliorated H_2_O_2_-induced ERK1/2 and p38 phosphorylation.

### Effects of oral KI Essence extract treatment on myelination in maternal immune activation offspring

**FIGURE 4:**
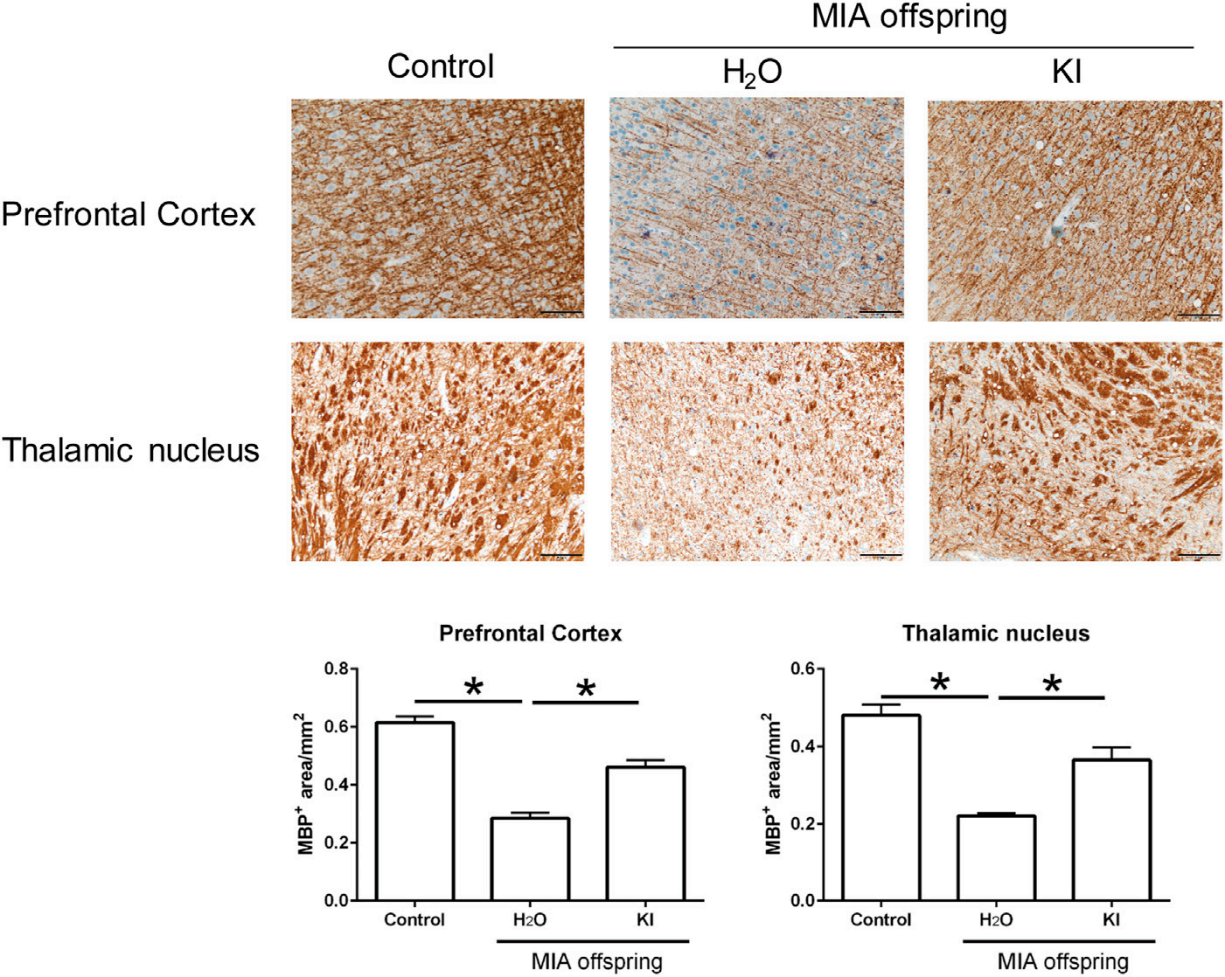
Amelioration of hypomyelination in maternal immune activation (MIA) offspring after oral KI Essence extract treatment. Myelin basic protein (MBP) expression levels in the prefrontal cortex and thalamic nucleus of control group, H_2_O-treated MIA offspring, and KI Essence extract–treated MIA offspring were detected through immunohistochemical staining. Quantification of MBP^+^ area in the prefrontal cortex and thalamic nucleus is presented in the bar graph ^∗^ *p* < 0.05; *n* = 3 for each group). All data are presented as means ± standard errors of means (scale bar: 100 µm).

Maternal LPS stimulation causes oxidative stress and hypomyelination in the prefrontal cortex and thalamus nucleus of MIA offspring (Wischhof et al., 2015; Simoes et al., 2018). In this study, to evaluate the effects of KI Essence extract treatment on the *in vivo* modulation of myelination in the rat brain, we examined myelination levels in MIA offspring brains after oral KI Essence extract treatment. Immunohistochemical staining revealed that the MBP^+^ area was smaller in the prefrontal cortex and thalamic nucleus of the MIA offspring (H_2_O treatment) than in the control rats; nevertheless, oral KI Essence extract treatment alleviated the loss of the MBP^+^ area in the MIA offspring (Figure 4). These results indicated that oral KI Essence extract treatment alleviated hypomyelination in MIA offspring.

### Effects of oral KI Essence extract treatment on microbiome profile in maternal immune activation offspring

**FIGURE 5:**
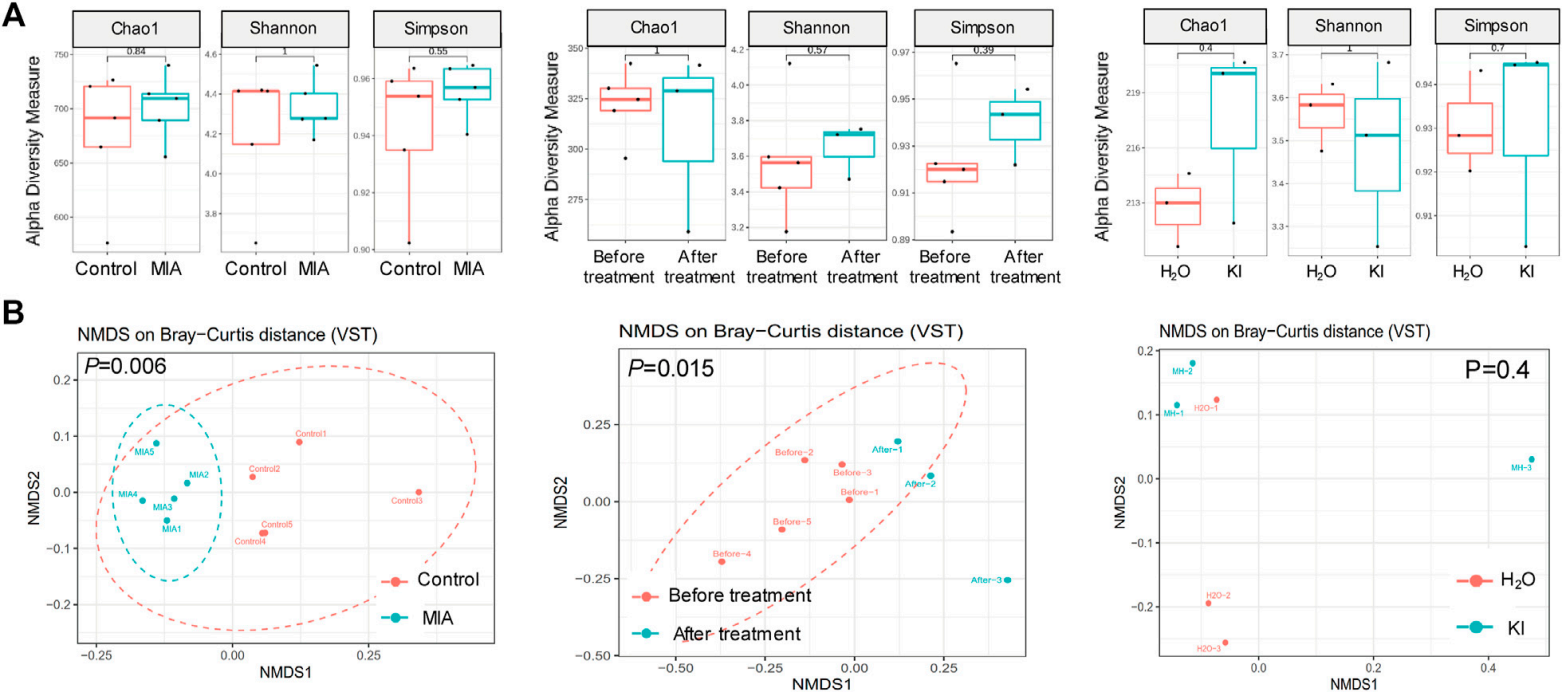
Modulation of fecal microbiome distribution in maternal immune activation (MIA) offspring after oral KI Essence extract treatment. Fecal microbiome profiling of male MIA offspring was performed through high-throughput 16S rRNA gene sequencing. **(A)** Alpha diversity of fecal microbiota and **(B)** principal coordinate analysis plots (as obtained through nonmetric multidimensional scaling with Bray–Curtis distance analysis) for control and MIA rats before and after H_2_O and KI Essence extract treatment. Permutational multivariate analysis of variance (vegan::adonis, 1000 permutations) revealed a significant difference in beta diversity, which was quantified using a betadisper (vegan::betadisper, 1000 permutations). Adonis and betadisper indices yielded *p* values of <0.05 and >0.05, respectively. *n* = 5 for control and MIA group; *n* = 5 for before treatment group; *n* = 3 for after treatment, H_2_O, and KI Essence extract treatment group.

**FIGURE 6:**
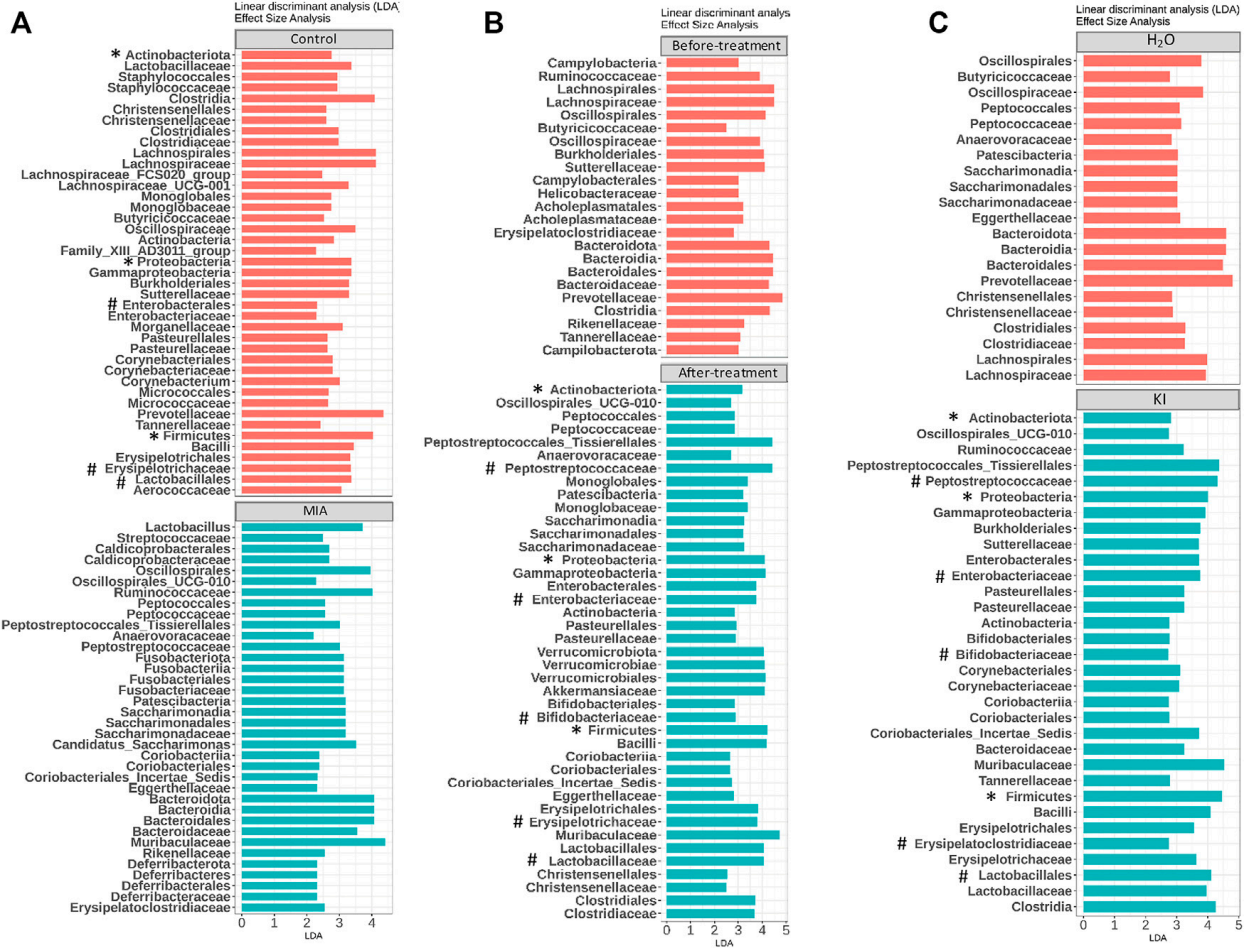
Modulation of microbiome profile in maternal immune activation (MIA) offspring after oral KI Essence extract treatment. Linear discriminant analyses (LDAs) for comparing **(A)** gut microbiota effect sizes of control and MIA offspring, **(B)** gut microbiota effect sizes of MIA offspring before and after KI Essence extract treatment, and **(C)** gut microbiota effect sizes of MIA offspring after H_2_O and KI Essence extract (KI) treatment. Significant biomarkers are defined as taxa with LDA score (log_10_) ≥ 2. ^∗^ and # indicated that abundance of bacterial species at the phylum and family levels was significantly different, respectively. *n* = 5 for control and MIA group; *n* = 5 for before treatment group; *n* = 3 for after treatment, H_2_O, and KI Essence extract treatment group.

Mushrooms contain bioactive ingredients that can modulate gut microbiota (Ma et al., 2021; Vamanu et al., 2021). LPS-induced MIA offspring exhibit a microbiome profile similar to that of humans with ASD (Lee et al., 2021). Therefore, we examined the effects of KI Essence extract treatment on the modulation of the microbiome profile in the examined MIA offspring. The alpha diversity of the fecal microbiota in the MIA offspring was similar to that of the control offspring (Figure 5A, left panel). Nonmetric multidimensional scaling (NMDS) was performed using the Bray–Curtis distance method, and the results indicated that the fecal microbiome profile of the MIA offspring was significantly different from that of the control offspring (Figure 5B, left panel). As presented in Figure 6A, significant differences were found in the abundance of microbial species between the control and MIA offspring. Compared with the MIA offspring, the control offspring had significantly higher LDA scores for Firmicutes*,* Proteobacteria, and Actinobacteriota bacteria at the phylum level. NMDS with Bray–Curtis distance analysis was subsequently performed, and the results indicated that after 2 weeks of oral KI Essence extract treatment, the fecal microbiome profile of the MIA offspring was significantly different from that of the MIA offspring (Figure 5B, middle panel); however, the differences in alpha diversity were nonsignificant (Figure 5A, middle panel). By contrast, the oral KI Essence extract–treated MIA offspring did not differ significantly from the H_2_O-treated male MIA offspring in terms of the beta diversity of their microbiota (Figure 5B, right panel). At the phylum level, the LDA scores for Firmicutes, Proteobacteria, and Actinobacteriota bacteria in the MIA offspring were higher after oral KI Essence extract treatment than before treatment (Figure 6B); these scores were also higher than those of the H_2_O-treated MIA offspring (Figure 6C). Similarly, at the family level, the LDA scores for Peptostreptococcaceae, Enterobacteriaceae, Erysipelotrichaceae, Lactobacillaceae, and Bifidobacteriaceae bacteria in the MIA offspring were higher after oral KI Essence extract treatment than before treatment (Figure 6B); these scores were also higher than those of the H_2_O-MIA offspring (Figure 6C). In summary, oral KI Essence extract treatment could modulate the dysbiotic microbiome profile of the MIA offspring.

### Effects of KI Essence extract treatment on lipopolysaccharide-stimulated microglia

**FIGURE 7:**
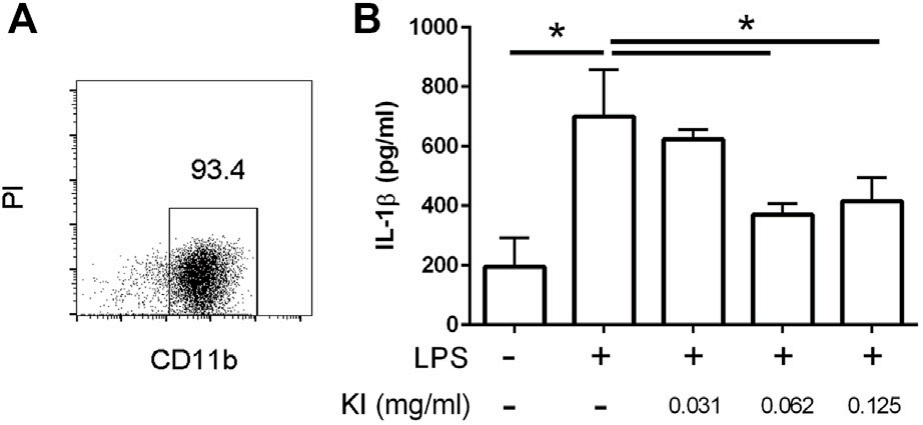
Inhibitory effect of KI Essence treatment on interleukin (IL)-1β production from lipopolysaccharide (LPS)-induced microglia activation. **(A)** Percentage of CD11b^+^ cells in enriched microglia cell culture. PI: propidium iodide. **(B)** Microglia treated with KI Essence (0, 0.031, 0.062, 0.125 mg/ml) in the presence of 250 ng/ml LPS for 24 h. Amount of IL-1β in culture supernatant was determined through enzyme-linked immunosorbent assay (*n* = 3; ^∗^ *p* < 0.05).

During the gestational stage, elevated IL-1β levels are associated with hypomyelination in LPS-induced MIA offspring (Rousset et al., 2006; Simoes et al., 2018; Chamera et al., 2020). LPS stimulation prompts IL-1β production in microglia (Kim et al., 2006). In this study, we observed that KI Essence extract treatment inhibited IL-1β production in LPS-activated microglia (Figure 7).

## Discussion

In the present study, we observed that the extract of the spleen-tonifying formula KI Essence promoted neuritogenesis activity, alleviated hypomyelination, and modulated the microbiome profile in MIA offspring. Supplementary Figure S4 presents a schematic of the workflow for the present study. Our results were as follows: 1) KI Essence extract treatment enhanced p-ERK expression to promote NGF-induced neuritogenesis and inhibited p-ERK expression to protect differentiated PC12 cells from H_2_O_2_-induced cell death. 2) The KI Essence extract demonstrated DPPH radical inhibition and reduced the peroxidation response in rat brain homogenates. 3) Oral KI Essence extract treatment alleviated prenatal LPS-induced hypomyelination in the prefrontal cortex and thalamic nucleus of the MIA offspring and increased the abundance of Peptostreptococcaceae, Enterobacteriaceae, Erysipelotrichaceae, Bifidobacteriaceae, and Lactobacillales bacteria in the offspring. This is the first study to demonstrate that treatment with a spleen-tonifying formula can promote neurite growth, alleviate oxidative stress, and alter the brain–gut–microbiota axis in MIA offspring.

Mushroom and traditional Chinese herbal extracts contain polysaccharides and nucleotides that can induce neuronal differentiation and promote antioxidant activity (Sabaratnam et al., 2013; Kozarski et al., 2015; Cor et al., 2018). Our three-dimensional HPLC fingerprint analysis revealed the presence of guanosine, uridine, and maslinic acid in the examined KI Essence extract (Supplementary Figure S1). Guanosine exhibits antioxidant activity and protects DNA from the oxidative damage induced by ROS (Gudkov et al., 2006). Uridine has been noted to enhance neurite outgrowth in NGF-differentiated PC12 cells (Pooler et al., 2005). Maslinic acid promotes synaptogenesis and axon growth (Qian et al., 2015). In the present study, the examined KI Essence extract increased ERK1/2 phosphorylation to promote neurite outgrowth in differentiated PC12 cells under NGF stimulation. We also observed that KI Essence extract treatment ameliorated the phosphorylation level of ERK1/2 in H_2_O_2_-treated PC12 cells. These contrasting effects on ERK phosphorylation in PC12 cells are attributable to the different components in KI Essence: maslinic acid and uridine in KI Essence may promote neurite outgrowth by upregulating ERK phosphorylation. Polysaccharides and guanosine in KI Essence can ameliorate H_2_O_2_-induced oxidative stress and thus inhibit H_2_O_2_-induced ERK phosphorylation. Our data indicated that p-p38 expression slightly increased after 30-min treatment with both KI Essence extract and H_2_O_2_, but it decreased after 60 min of this treatment. This finding suggests that KI Essence components enhance p-p38 expression after 30 min of KI Essence extract treatment in the presence of H_2_O_2_ stimulation. We also noted that although H_2_O_2_ stimulation caused the p-p38 expression signal to peak after 1 h of treatment, the KI Essence extract exhibited antioxidant activity to ameliorate the effects of H_2_O_2_ stimulation on p-p38 expression. Thus, our results demonstrate that KI Essence can promote neurite outgrowth and antioxidant activity.

Prenatal LPS stimulation causes oxidative stress (including ROS generation and peroxisomal dysfunction), inflammation, and hypomyelination in MIA offspring (Arsenault et al., 2014; Maas et al., 2017). (Yui et al., 2020) also reported that lipid peroxidation levels are higher in patients with ASD than in healthy controls. Oxidative stress inhibits oligodendrocyte maturation by inhibiting differentiation-related gene expression; thus, lipid peroxidation stress may affect the maturation of oligodendrocytes (French et al., 2009; Chew et al., 2020). LPS-activated microglia cause oligodendrocyte progenitor cell death, reducing the number of mature myelinating oligodendrocytes (Pang et al., 2010). The elevated levels of the inflammatory cytokine IL-1β during the gestational stage is associated with hypomyelination in LPS-induced MIA offspring with ASD-like behavior (Rousset et al., 2006; Simoes et al., 2018; Chamera et al., 2020). In the present study, the examined KI Essence extract demonstrated DPPH radical scavenging activity, ameliorated lipid peroxidation in rat brain homogenates, and inhibited IL-1β production in LPS-activated microglia. These findings suggest that oral KI Essence extract treatment inhibits prenatal LPS-induced oxidative stress and microglia activation in MIA offspring, thereby alleviating hypomyelination in the prefrontal cortex and thalamic nucleus of the offspring.

Microbial dysbiosis is correlated with behavioral abnormalities and neuropathology (Vuong and Hsiao, 2017). Mushrooms are functional foods containing various biologically active compounds that can mitigate microbial dysbiosis (Cheung et al., 2020; Vamanu et al., 2021). Gut microbiota transplantation (primarily with a mixture of bifidobacteria*,* streptococci, and lactobacilli) is a therapeutic method used to alter microbiome profiles and improve neurobehavioral symptoms (Kang et al., 2017; Fattorusso et al., 2019; Chen et al., 2020; Abuaish et al., 2021). In this study, the low abundance of Enterobacteriaceae, Actinobacteria, Peptostreptococcaceae, and Erysipelotrichaceae that was observed in the LPS-induced MIA rat offspring is consistent with the microbiome profile in humans with ASD (Rosenfeld, 2015; Liu et al., 2019; Xu et al., 2019). Patients with ASD exhibit a low abundance of Bifidobacteriaceae family bacteria (phylum Actinobacteriota) (Finegold et al., 2010; Xu et al., 2019). We observed that oral KI Essence extract treatment increased the number of Enterobacteriaceae, Actinobacteria, Peptostreptococcaceae, Erysipelotrichaceae, and *Bifidobacterium* bacteria in the MIA offspring. Current psychopharmacological treatments include psychotropic medications and dietary supplements with antioxidant activity (Aishworiya et al., 2022). The intake of supplements with antioxidant activity leads to the reduction of ROS levels and the upregulation of genes involved in detoxification and neuroprotection in the central nervous system (Pangrazzi et al., 2020a). Our results suggest that KI Essence has the potential to serve as an ancillary treatment for alleviating oxidative stress and dysbiosis.

## Conclusion

The KI Essence extract was noted to have dual effects in an NGF-differentiated PC12 cell model: neurite outgrowth and antioxidant properties promotion through ERK1/2 signaling pathway activation and inhibition, respectively. Because of its antioxidant and radical scavenging properties, this extract could also alleviate MIA-induced oxidative stress and prevent myelin loss in MIA offspring. Oral KI Essence extract treatment increased the deficits in the microbial species, including those from Enterobacteriaceae*,* Actinobacteria*,* Peptostreptococcaceae, Erysipelotrichaceae, and *Bifidobacterium*; this result is similar to that observed in patients with ASD.

This study highlights the relationship among the microbiome, immune system, and central nervous system, which is crucial in the etiopathophysiology of mental disorders. Furthermore, KI Essence extract treatment can alter abnormal brain–gut–microbiota axis phenotypes. Nevertheless, three issues as limitations of this study remain unresolved. First, DPPH is a chemical test and may not accurately measure the radical scavenging effects of KI Essence extracts. Second, the single dose study was used in the study. Several doses should have been used to get a more accurate conclusion. Third, whether KI Essence can alleviate mental disorder symptoms, such as social behavior deficit, and whether different oral doses of KI Essence have varied effects on MIA offspring warrant further research.

## Data Availability

The original contributions presented in the study are included in the article/Supplementary Material, further inquiries can be directed to the corresponding author.

## References

[B1] Abuaish S. Al-Otaibi N. M. Abujamel T. S. Alzahrani S. A. Alotaibi S. M. Alshawakir Y. A. (2021). Fecal transplant and Bifidobacterium treatments modulate gut Clostridium bacteria and rescue social impairment and hippocampal BDNF expression in a rodent model of autism. Brain Sci. 11, 1038. 10.3390/brainsci11081038 34439657 PMC8391663

[B2] Aishworiya R. Valica T. Hagerman R. Restrepo B. (2022). An update on psychopharmacological treatment of autism spectrum disorder. Neurotherapeutics 19, 248–262. 10.1007/s13311-022-01183-1 35029811 PMC9130393

[B3] Arsenault D. St-Amour I. Cisbani G. Rousseau L. S. Cicchetti F. (2014). The different effects of LPS and poly I:C prenatal immune challenges on the behavior, development and inflammatory responses in pregnant mice and their offspring. Brain Behav. Immun. 38, 77–90. 10.1016/j.bbi.2013.12.016 24384468

[B4] Bang M. Lee S. H. Cho S.-H. Yu S.-A. Kim K. Lu H. Y. (2017). Herbal medicine treatment for children with autism spectrum disorder: A systematic review. Evid. Based. Complement. Altern. Med. 2017, 8614680. 10.1155/2017/8614680 PMC5448044 28592982

[B5] Callahan B. J. Mcmurdie P. J. Rosen M. J. Han A. W. Johnson A. J. Holmes S. P. (2016a). DADA2: High-resolution sample inference from Illumina amplicon data. Nat. Methods 13, 581–583. 10.1038/nmeth.3869 27214047 PMC4927377

[B6] Callahan B. J. Sankaran K. Fukuyama J. A. Mcmurdie P. J. Holmes S. P. (2016b). Bioconductor workflow for microbiome data analysis: From raw reads to community analyses. F1000Res. 5, 1492. 10.12688/f1000research.8986.2 27508062 PMC4955027

[B7] Chamera K. Kotarska K. Szuster-Gluszczak M. Trojan E. Skorkowska A. Pomierny B. (2020). The prenatal challenge with lipopolysaccharide and polyinosinic:polycytidylic acid disrupts cx3cl1-cx3cr1 and cd200-cd200r signalling in the brains of male rat offspring: a link to schizophrenia-like behaviours. J. Neuroinflammation 17, 247. 10.1186/s12974-020-01923-0 32829711 PMC7444338

[B8] Chauhan A. Chauhan V. (2006). Oxidative stress in autism. Pathophysiology 13, 171–181. 10.1016/j.pathophys.2006.05.007 16766163

[B9] Chen J. Bittinger K. Charlson E. S. Hoffmann C. Lewis J. Wu G. D. (2012). Associating microbiome composition with environmental covariates using generalized UniFrac distances. Bioinformatics 28, 2106–2113. 10.1093/bioinformatics/bts342 22711789 PMC3413390

[B10] Chen K. Fu Y. Wang Y. Liao L. Xu H. Zhang A. (2020). Therapeutic effects of the *in vitro* cultured human gut microbiota as transplants on altering gut microbiota and improving symptoms associated with autism spectrum disorder. Microb. Ecol. 80, 475–486. 10.1007/s00248-020-01494-w 32100127

[B11] Cheung M. K. Yue G. G. L. Chiu P. W. Y. Lau C. B. S. (2020). A review of the effects of natural compounds, medicinal plants, and mushrooms on the gut microbiota in colitis and cancer. Front. Pharmacol. 11, 744. 10.3389/fphar.2020.00744 32499711 PMC7243258

[B12] Chew H. Solomon V. A. Fonteh A. N. (2020). Involvement of lipids in alzheimer's disease pathology and potential therapies. Front. Physiol. 11, 598. 10.3389/fphys.2020.00598 32581851 PMC7296164

[B13] Cor D. Knez Z. Knez Hrncic M. (2018). Antitumour, antimicrobial, antioxidant and antiacetylcholinesterase effect of Ganoderma lucidum terpenoids and polysaccharides: A review. Molecules 23, E649. 10.3390/molecules23030649 29534044 PMC6017764

[B14] Diallo I. Boudard F. Morel S. Vitou M. Guzman C. Saint N. (2020). Antioxidant and anti-inflammatory potential of shiitake culinary-medicinal mushroom, lentinus edodes (agaricomycetes), sporophores from various culture conditions. Int. J. Med. Mushrooms 22, 535–546. 10.1615/IntJMedMushrooms.2020034864 32865895

[B15] Fattorusso A. Di Genova L. Dell'isola G. B. Mencaroni E. Esposito S. (2019). Autism Spectrum Disorders and the Gut Microbiota, Nutrients 11, 521. 10.3390/nu11030521 PMC6471505 30823414

[B16] Finegold S. M. Dowd S. E. Gontcharova V. Liu C. Henley K. E. Wolcott R. D. (2010). Pyrosequencing study of fecal microflora of autistic and control children. Anaerobe 16, 444–453. 10.1016/j.anaerobe.2010.06.008 20603222

[B17] French H. M. Reid M. Mamontov P. Simmons R. A. Grinspan J. B. (2009). Oxidative stress disrupts oligodendrocyte maturation. J. Neurosci. Res. 87, 3076–3087. 10.1002/jnr.22139 19479983 PMC3138415

[B18] Graciarena M. Seiffe A. Nait-Oumesmar B. Depino A. M. (2018). Hypomyelination and oligodendroglial alterations in a mouse model of autism spectrum disorder. Front. Cell. Neurosci. 12, 517. 10.3389/fncel.2018.00517 30687009 PMC6338056

[B19] Greenwood M. T. (2017). Dysbiosis, spleen Qi, phlegm, and complex difficulties. Med. Acupunct. 29, 128–137. 10.1089/acu.2017.1226 28736589 PMC5512334

[B20] Gudkov S. V. Shtarkman I. N. Smirnova V. S. Chernikov A. V. Bruskov V. I. (2006). Guanosine and inosine display antioxidant activity, protect DNA *in vitro* from oxidative damage induced by reactive oxygen species, and serve as radioprotectors in mice. Radiat. Res. 165, 538–545. 10.1667/RR3552.1 16669708

[B21] Hughes H. K. Rose D. Ashwood P. (2018). The gut microbiota and dysbiosis in autism spectrum disorders. Curr. Neurol. Neurosci. Rep. 18, 81. 10.1007/s11910-018-0887-6 30251184 PMC6855251

[B22] Kang D. W. Adams J. B. Gregory A. C. Borody T. Chittick L. Fasano A. (2017). Microbiota transfer therapy alters gut ecosystem and improves gastrointestinal and autism symptoms: an open-label study. Microbiome 5, 10. 10.1186/s40168-016-0225-7 28122648 PMC5264285

[B23] Kim J. H. Ha H. C. Lee M. S. Kang J. I. Kim H. S. Lee S. Y. (2007). Effect of Tremella fuciformis on the neurite outgrowth of PC12h cells and the improvement of memory in rats. Biol. Pharm. Bull. 30, 708–714. 10.1248/bpb.30.708 17409507

[B24] Kim Y. J. Hwang S. Y. Oh E. S. Oh S. Han I. O. (2006). IL-1beta, an immediate early protein secreted by activated microglia, induces iNOS/NO in C6 astrocytoma cells through p38 MAPK and NF-kappaB pathways. J. Neurosci. Res. 84, 1037–1046. 10.1002/jnr.21011 16881054

[B25] Kozarski M. Klaus A. Jakovljevic D. Todorovic N. Vunduk J. Petrovic P. (2015). Antioxidants of edible mushrooms. Molecules 20, 19489–19525. 10.3390/molecules201019489 26516828 PMC6331815

[B26] Lee G. A. Lin Y. K. Lai J. H. Lo Y. C. Yang Y. S. H. Ye S. Y. (2021). Maternal immune activation causes social behavior deficits and hypomyelination in male rat offspring with an autism-like microbiota profile. Brain Sci. 11, 1085. 10.3390/brainsci11081085 34439704 PMC8391334

[B27] Lee S. E. Ju E. M. Kim J. H. (2002). Antioxidant activity of extracts from Euryale ferox seed. Exp. Mol. Med. 34, 100–106. 10.1038/emm.2002.15 12085984

[B28] Li H. Lee H. S. Kim S. H. Moon B. Lee C. (2014). Antioxidant and anti-inflammatory activities of methanol extracts of Tremella fuciformis and its major phenolic acids. J. Food Sci. 79, C460–C468. 10.1111/1750-3841.12393 24547933

[B29] Li W. Chen M. Feng X. Song M. Shao M. Yang Y. (2021). Maternal immune activation alters adult behavior, intestinal integrity, gut microbiota and the gut inflammation. Brain Behav. 11, e02133. 10.1002/brb3.2133 33793085 PMC8119836

[B30] Liu S. Li E. Sun Z. Fu D. Duan G. Jiang M. (2019). Altered gut microbiota and short chain fatty acids in Chinese children with autism spectrum disorder. Sci. Rep. 9, 287. 10.1038/s41598-018-36430-z 30670726 PMC6342986

[B31] Lu Y. Guo S. Zhang F. Yan H. Qian D. W. Wang H. Q. (2019). Comparison of functional components and antioxidant activity of Lycium barbarum L. Fruits from different regions in China. Molecules 24, E2228. 10.3390/molecules24122228 31207958 PMC6632000

[B32] Ma G. Du H. Hu Q. Yang W. Pei F. Xiao H. (2021). Health benefits of edible mushroom polysaccharides and associated gut microbiota regulation. Crit. Rev. Food Sci. Nutr., 1–18. 10.1080/10408398.2021.1903385 33792430

[B33] Maas D. A. Valles A. Martens G. J. M. (2017). Oxidative stress, prefrontal cortex hypomyelination and cognitive symptoms in schizophrenia. Transl. Psychiatry 7, e1171. 10.1038/tp.2017.138 28934193 PMC5538118

[B34] Masuko T. Minami A. Iwasaki N. Majima T. Nishimura S. Lee Y. C. (2005). Carbohydrate analysis by a phenol-sulfuric acid method in microplate format. Anal. Biochem. 339, 69–72. 10.1016/j.ab.2004.12.001 15766712

[B35] Matkowski A. Jamiolkowska-Kozlowska W. Nawrot I. (2013). Chinese medicinal herbs as source of antioxidant compounds--where tradition meets the future. Curr. Med. Chem. 20, 984–1004. 10.2174/0929867311320080003 23210784

[B36] Mcmurdie P. J. Holmes S. (2013). phyloseq: an R package for reproducible interactive analysis and graphics of microbiome census data. PLoS One 8, e61217. 10.1371/journal.pone.0061217 23630581 PMC3632530

[B37] Nguyen H. T. N. Kato H. Masuda K. Yamaza H. Hirofuji Y. Sato H. (2018). Impaired neurite development associated with mitochondrial dysfunction in dopaminergic neurons differentiated from exfoliated deciduous tooth-derived pulp stem cells of children with autism spectrum disorder. Biochem. Biophys. Rep. 16, 24–31. 10.1016/j.bbrep.2018.09.004 30258988 PMC6153399

[B38] Nie A. Chao Y. Zhang X. Jia W. Zhou Z. Zhu C. (2020). Phytochemistry and pharmacological activities of Wolfiporia cocos (F.A. Wolf) ryvarden & gilb. Front. Pharmacol. 11, 505249. 10.3389/fphar.2020.505249 33071776 PMC7533546

[B39] Nitschke A. Deonandan R. Konkle A. T. (2020). The link between autism spectrum disorder and gut microbiota: A scoping review. Autism 24, 1328–1344. 10.1177/1362361320913364 32340474

[B40] Olugbami J. O. Gbadegesin M. A. Odunola O. A. (2014). *In vitro* evaluation of the antioxidant potential, phenolic and flavonoid contents of the stem bark ethanol extract of Anogeissus leiocarpus. Afr. J. Med. Med. Sci. 43, 101–109. 26681826 PMC4679201

[B41] Pang Y. Campbell L. Zheng B. Fan L. Cai Z. Rhodes P. (2010). Lipopolysaccharide-activated microglia induce death of oligodendrocyte progenitor cells and impede their development. Neuroscience 166, 464–475. 10.1016/j.neuroscience.2009.12.040 20035837

[B42] Pangrazzi L. Balasco L. Bozzi Y. (2020a). Natural Antioxidants: A Novel Therapeutic Approach to Autism Spectrum Disorders? Antioxidants (Basel) 9, 1186. 10.3390/antiox9121186 PMC7761361 33256243

[B43] Pangrazzi L. Balasco L. Bozzi Y. (2020b). Oxidative stress and immune system dysfunction in autism spectrum disorders. Int. J. Mol. Sci. 21, E3293. 10.3390/ijms21093293 32384730 PMC7247582

[B44] Peralta-Marzal L. N. Prince N. Bajic D. Roussin L. Naudon L. Rabot S. (2021). The impact of gut microbiota-derived metabolites in autism spectrum disorders. Int. J. Mol. Sci. 22, 10052. 10.3390/ijms221810052 34576216 PMC8470471

[B45] Pi K. Lee K. (2017). Prunus mume extract exerts antioxidant activities and suppressive effect of melanogenesis under the stimulation by alpha-melanocyte stimulating hormone in B16-F10 melanoma cells. Biosci. Biotechnol. Biochem. 81, 1883–1890. 10.1080/09168451.2017.1365591 28831862

[B46] Pooler A. M. Guez D. H. Benedictus R. Wurtman R. J. (2005). Uridine enhances neurite outgrowth in nerve growth factor-differentiated PC12 [corrected]. Neuroscience 134, 207–214. 10.1016/j.neuroscience.2005.03.050 15939540

[B47] Qian Y. Huang M. Guan T. Chen L. Cao L. Han X. J. (2015). Maslinic acid promotes synaptogenesis and axon growth via Akt/GSK-3β activation in cerebral ischemia model. Eur. J. Pharmacol. 764, 298–305. 10.1016/j.ejphar.2015.07.028 26172083

[B48] Quast C. Pruesse E. Yilmaz P. Gerken J. Schweer T. Yarza P. (2013). The SILVA ribosomal RNA gene database project: Improved data processing and web-based tools. Nucleic Acids Res. 41, D590–D596. 10.1093/nar/gks1219 23193283 PMC3531112

[B49] Rajaei A. Salarbashi D. Asrari N. Fazly Bazzaz B. S. Aboutorabzade S. M. Shaddel R. (2021). Antioxidant, antimicrobial, and cytotoxic activities of extracts from the seed and pulp of Jujube (Ziziphus jujuba) grown in Iran. Food Sci. Nutr. 9, 682–691. 10.1002/fsn3.2031 33598153 PMC7866595

[B50] Ranogajec A. Beluhan S. Smit Z. (2010). Analysis of nucleosides and monophosphate nucleotides from mushrooms with reversed-phase HPLC. J. Sep. Sci. 33, 1024–1033. 10.1002/jssc.200900516 20175083

[B51] Rezatabar S. Karimian A. Rameshknia V. Parsian H. Majidinia M. Kopi T. A. (2019). RAS/MAPK signaling functions in oxidative stress, DNA damage response and cancer progression. J. Cell. Physiol. 234, 14951–14965. 10.1002/jcp.28334 30811039

[B52] Rios J. L. (2011). Chemical constituents and pharmacological properties of Poria cocos. Planta Med. 77, 681–691. 10.1055/s-0030-1270823 21347995

[B53] Rosenfeld C. S. (2015). Microbiome disturbances and autism spectrum disorders. Drug Metab. Dispos. 43, 1557–1571. 10.1124/dmd.115.063826 25852213

[B54] Rousset C. I. Chalon S. Cantagrel S. Bodard S. Andres C. Gressens P. (2006). Maternal exposure to LPS induces hypomyelination in the internal capsule and programmed cell death in the deep gray matter in newborn rats. Pediatr. Res. 59, 428–433. 10.1203/01.pdr.0000199905.08848.55 16492984

[B55] Sabaratnam V. Kah-Hui W. Naidu M. Rosie David P. (2013). Neuronal health - can culinary and medicinal mushrooms help? J. Tradit. Complement. Med. 3, 62–68. 10.4103/2225-4110.106549 24716157 PMC3924982

[B56] Schliep K. P. (2011). phangorn: phylogenetic analysis in R. Bioinformatics 27, 592–593. 10.1093/bioinformatics/btq706 21169378 PMC3035803

[B57] Segata N. Izard J. Waldron L. Gevers D. Miropolsky L. Garrett W. S. (2011). Metagenomic biomarker discovery and explanation. Genome Biol. 12, R60. 10.1186/gb-2011-12-6-r60 21702898 PMC3218848

[B58] Simoes L. R. Sangiogo G. Tashiro M. H. Generoso J. S. Faller C. J. Dominguini D. (2018). Maternal immune activation induced by lipopolysaccharide triggers immune response in pregnant mother and fetus, and induces behavioral impairment in adult rats. J. Psychiatr. Res. 100, 71–83. 10.1016/j.jpsychires.2018.02.007 29494891

[B59] Svoboda E. (2020). Could the gut microbiome be linked to autism? Nature 577, S14-S15–S15. 10.1038/d41586-020-00198-y 31996829

[B60] Vamanu E. Dinu L. D. Pelinescu D. R. Gatea F. (2021). Therapeutic properties of edible mushrooms and herbal teas in gut microbiota modulation. Microorganisms 9, 1262. 10.3390/microorganisms9061262 34200833 PMC8230450

[B61] Vuong H. E. Hsiao E. Y. (2017). Emerging roles for the gut microbiome in autism spectrum disorder. Biol. Psychiatry 81, 411–423. 10.1016/j.biopsych.2016.08.024 27773355 PMC5285286

[B62] Wang X. Wang Z. Yao Y. Li J. Zhang X. Li C. (2011). Essential role of ERK activation in neurite outgrowth induced by alpha-lipoic acid. Biochim. Biophys. Acta 1813, 827–838. 10.1016/j.bbamcr.2011.01.027 21295083

[B63] Wiatrak B. Kubis-Kubiak A. Piwowar A. Barg E. (2020). PC12 cell line: Cell types, coating of culture vessels, differentiation and other culture conditions. Cells 9, 958. 10.3390/cells9040958 PMC7227003 32295099

[B64] Wischhof L. Irrsack E. Osorio C. Koch M. (2015). Prenatal LPS-exposure--a neurodevelopmental rat model of schizophrenia--differentially affects cognitive functions, myelination and parvalbumin expression in male and female offspring. Prog. Neuropsychopharmacol. Biol. Psychiatry 57, 17–30. 10.1016/j.pnpbp.2014.10.004 25455585

[B65] Wu D.-T. Liu W. Han Q.-H. Wang P. Xiang X.-R. Ding Y. (2019). Extraction optimization, structural characterization, and antioxidant activities of polysaccharides from Cassia seed (Cassia obtusifolia). Molecules 24, 2817. 10.3390/molecules24152817 PMC6696105 31382366

[B66] Xu M. Xu X. Li J. Li F. (2019). Association between gut microbiota and autism spectrum disorder: A systematic review and meta-analysis. Front. Psychiatry 10, 473. 10.3389/fpsyt.2019.00473 31404299 PMC6673757

[B67] Xu X. Yang J. Ning Z. Zhang X. (2015). Lentinula edodes-derived polysaccharide rejuvenates mice in terms of immune responses and gut microbiota. Food Funct. 6, 2653–2663. 10.1039/c5fo00689a 26135107

[B68] Xu Y. Xie L. Zhang Z. Zhang W. Tang J. He X. (2021). Tremella fuciformis polysaccharides inhibited colonic inflammation in dextran sulfate sodium-treated mice via Foxp3+ T cells, gut microbiota, and bacterial metabolites. Front. Immunol. 12, 648162. 10.3389/fimmu.2021.648162 33868283 PMC8049506

[B69] Yuan F. Gao Z. Liu W. Li H. Zhang Y. Feng Y. (2019). Characterization, antioxidant, anti-aging and organ protective effects of sulfated polysaccharides from Flammulina velutipes. Molecules 24, E3517. 10.3390/molecules24193517 31569331 PMC6803911

[B70] Yui K. Imataka G. Sasaki H. Shiroki R. (2020). The role of lipid peroxidation in individuals with autism spectrum disorders. Metab. Brain Dis. 35, 1101–1108. 10.1007/s11011-020-00585-4 32643093

[B71] Zou Y. T. Zhou J. Wu C. Y. Zhang W. Shen H. Xu J. D. (2021). Protective effects of Poria cocos and its components against cisplatin-induced intestinal injury. J. Ethnopharmacol. 269, 113722. 10.1016/j.jep.2020.113722 33352240

