# Supplementary material for: KI Essence extract (a spleen-tonifying formula) promotes neurite outgrowth, alleviates oxidative stress and hypomyelination, and modulates microbiome in maternal immune activation offspring
Source: Front Pharmacol. 2022 Aug 25;13:964255. doi: 10.3389/fphar.2022.964255 (PMC9453593; doi:10.3389/fphar.2022.964255)
Supplement: Supplementary file 1 [file DataSheet1.PDF]

Supplementary Material

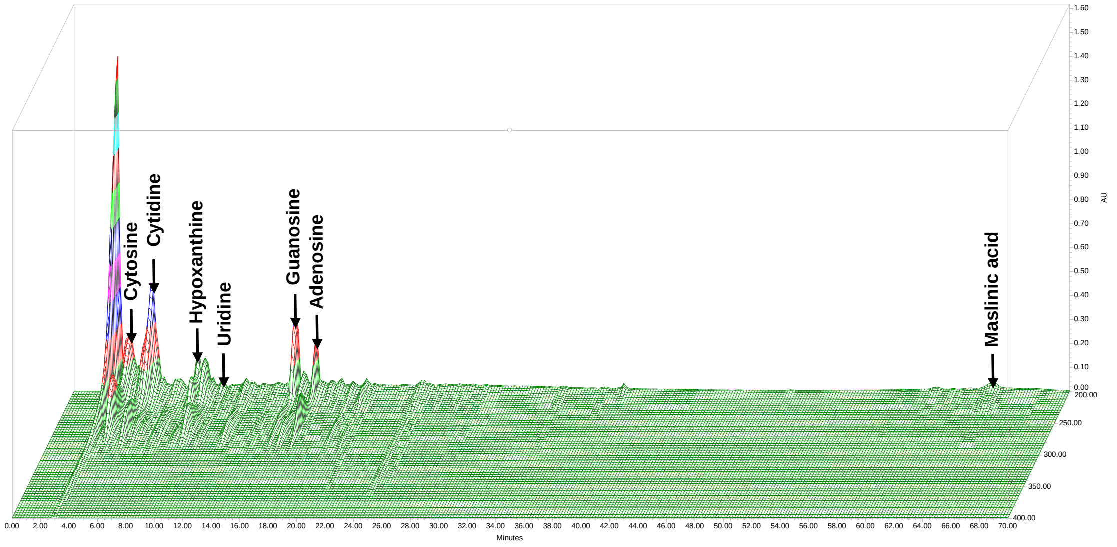

Figure 1. Qualitative analyses of KI Essence constituents by using high-performance liquid chromatography fingerprint analysis. A range of ultraviolet (200-400nm) chromatogram of KI Essence was shown. Cytosine, with a retention time of 4.1 min; Cytidine with a retention time of 5.9 min; Hypoxanthine with a retention time of 9.2 min; Uridine with a retention time of 11.9 min; Guanosine with a retention time of 15.8 min; Adenosine with a retention time of 17.1 min; Maslinic acid with a retention time of 65.2 min.

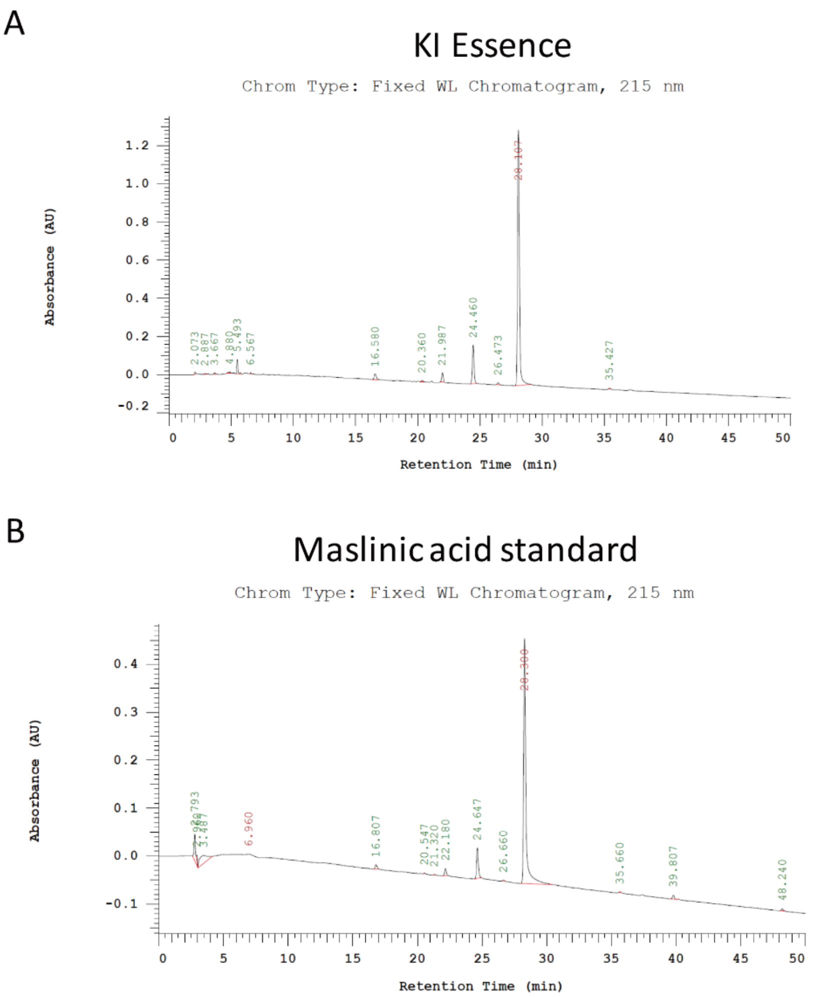

Figure 2. Results of qualitative analyses of KI Essence constituents by using high-performance liquid chromatography. (A) An ultraviolet chromatogram (215 nm) of the maslinic acid in KI Essence (10 mg/mL) with a retention time of 28.107 min, and (B) maslinic acid standard (50 µg/mL) with a retention time 28.300 min.

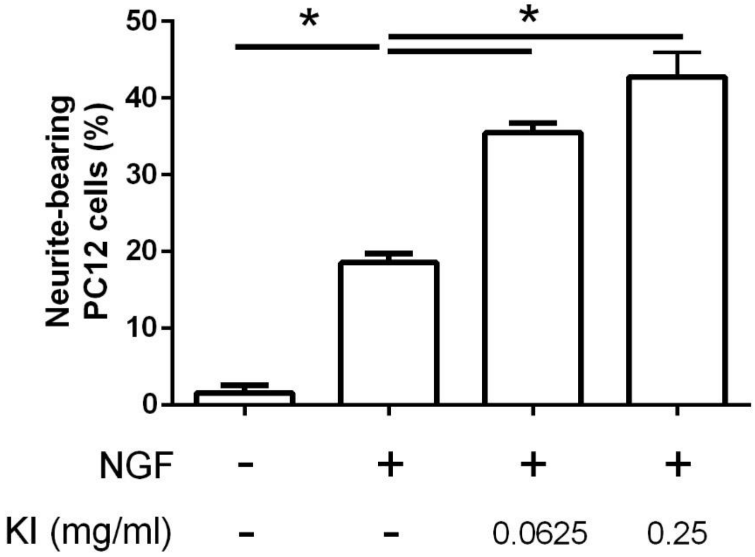

Figure 3. KI Essence extract enhanced neurite growth. PC12 cells were cotreated with KI Essence extract (0.0625 and 0.25 mg/ml) and NGF for 2 days, and percentage of neurite-bearing cells on day 2 was assessed. Data are expressed as means ± standard errors of means (SEMs) (*P < 0.05).

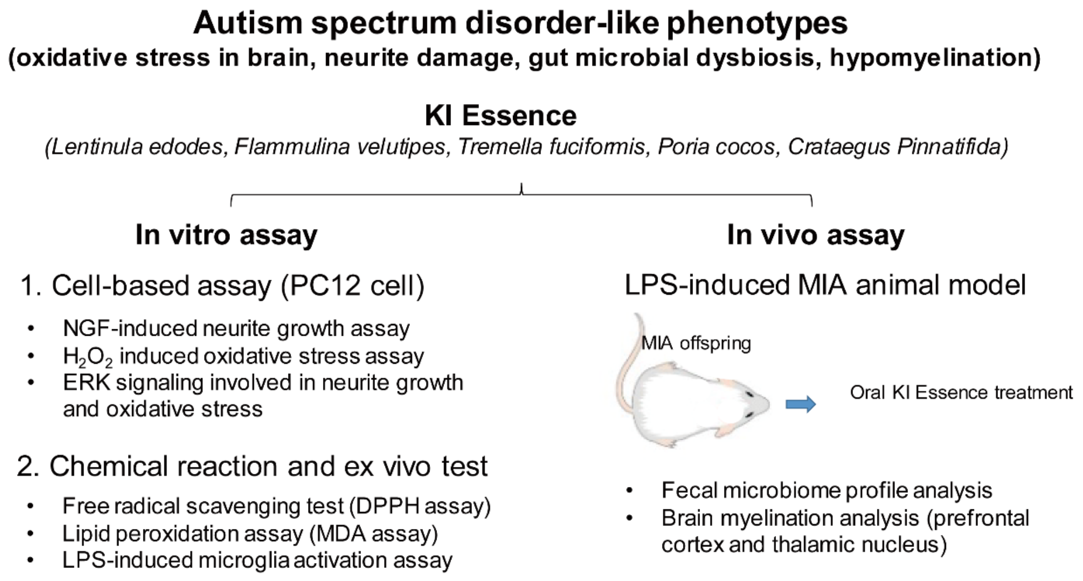

Figure 4. A workflow diagram of this study.

Table 1. Information of raw materials and their weight percentage in KI Essence preparation

Material	Fresh /dry material	Part of mushroom, herbs, organism	Origin	*Weight percentage (%)
Lentinula edodes	Dry	Fruiting body	China	10.1
Flammulina velutipes	Fresh	Fruiting body	Taiwan	12.12
Wolfiporia extensa	Dry	Sclerotium	China	12.12
Tremella fuciformis	Dry	Fruiting body	China	5.05
Crataegus Pinnatifida	Dry	Fruit	China	5.05
Lycium barbarum	Dry	Fruit	China	15.15
Senna obtusifolia	Dry	Fruit	China	15.15
Euryale ferox Salisb	Dry	Semen	China	5.05
Ziziphus Jujuba	Dry	Fruit	China	5.05
Prunus Mume	Dry	Fruit	China	3.03
Ostreae gigas	Dry	Concha	China	12.12

*Raw material weight percentage for preparation of KI Essence extract
